# Role of microRNAs in schistosomes and schistosomiasis

**DOI:** 10.3389/fcimb.2014.00165

**Published:** 2014-11-11

**Authors:** Lihui Zhu, Jinming Liu, Guofeng Cheng

**Affiliations:** Shanghai Veterinary Research Institute, Chinese Academy of Agricultural Sciences, Key Laboratory of Animal Parasitology, Ministry of AgricultureShanghai, China

**Keywords:** schistosome, microRNA, development, pathogenesis, biomarkers, schistosomiasis

## Abstract

Schistosomes, a class of parasitic trematode worms, cause schistosomiasis. Accumulating evidence suggests that microRNAs (miRNAs)—small, non-coding RNAs that are known to play critical regulatory roles in many organisms—may be involved in schistosome development and sexual maturation, as well as the pathogenesis of schistosomiasis. Schistosoma miRNAs, such as Bantam and miR-10, may be involved in the pathological processes of schistosomiasis, and recent studies suggest that schistosome-specific miRNAs (e.g., Bantam, miR-3479-3p) in the bloodstream of a final host could be used as biomarkers for schistosomiasis diagnosis. Furthermore, aberrant miRNAs, such as miR-223 and miR-454, can be produced by a host in response to schistosome infection, and these miRNAs may contribute to the pathogenesis of schistosomiasis-associated liver injury. Here, we summarize recent progress evaluating the relationship between schistosome miRNAs and schistosomiasis and discuss how these miRNAs can mediate the pathogenesis of schistosomiasis and be used as biomarkers for schistosomiasis diagnosis.

## Introduction

Schistosomiasis is a parasitic disease found worldwide and is caused primarily by worms of the genus *Schistosoma*. The main disease-causing species are *S. haematobium, S. mansoni, and S. japonicum*. Over 200 million people are infected with this parasite worldwide, leading to 300,000 deaths annually, mostly in the developing world (Colley et al., 2014). In China, schistosomiasis japonica is a serious public health problem (Zhou et al., 2005a,b). Unfortunately, there is no available vaccine for schistosomiasis. Praziquantel is the only drug that is widely used to treat schistosomiasis, but it is ineffective in preventing reinfection and there are concerns regarding the development of praziquantel resistance following large-scale and repeated use (Bergquist et al., 2008; Wang et al., 2012b). Consequently, a deeper understanding of the mechanisms of schistosome development and the pathogenesis of schistosomiasis may aid in the development of novel strategies to control this disease.

MicroRNAs (miRNAs), a class of small non-coding RNAs, can regulate a wide range of pivotal biological processes such as development, cell proliferation and differentiation, cell death, metabolism, and signal transduction in many organisms (Ambros, 2003; Carrington and Ambros, 2003; Miska, 2005). Schistosomes have a complex developmental cycle with diverse life stages, which suggests that gene expression in these organisms is regulated accurately and precisely (Gomes et al., 2009; Zhou et al., 2009; De Souza Gomes et al., 2011). Since miRNAs act as critical post-transcriptional regulators in many organisms, studies have been actively carried out to determine the role miRNAs play in schistosomes. These studies support our notion that miRNAs could be important regulators for schistosome development and potential therapeutic targets against schistosomiasis (Cheng and Jin, 2012). In addition, recent findings have provided new information regarding the roles of miRNAs in schistosome development and the pathogenesis of schistosomiasis. Here, we summarize recent research evaluating miRNAs in schistosomes and explore how schistosome miRNAs can serve as mediators of the pathogenesis of schistosomiasis. In addition, we also discuss the use of miRNAs as biomarkers for schistosomiasis diagnosis.

## Identification of schistosome miRNAs

To date, 79 mature miRNAs in *S. japonicum* and 225 mature miRNAs in *S. mansoni* have been documented in miRBase (Version 21). Although schistosome miRNAs have been summarized previously (Cheng and Jin, 2012), recent research has further investigated miRNA profiles in schistosomes, the results of these studies are summarized in Table 1.

**Table 1:** **Updated list of miRNAs identified in schistosomes**.

**Species**	**Conserved miRNAs**	**Species-specific miRNAs**	**Stages**	**Methods**	**References**
*S. japonicum*	65	213	L, H, M, F, E	Deep sequencing, Northern blotting, qRT-PCR	Cai et al., 2011
	4	1	A	Deep sequencing, qRT-PCR, semi-quantitative RT-PCR	Cheng et al., 2013
*S. mansoni*	61	48	–	Bioinformatics	De Souza Gomes et al., 2011
	26	71	A	Deep sequencing	Marco et al., 2013

A, adult worms (mixed males and females); L, lung-stage schistosomula; F, adult females; E, eggs; H, hepatic schistosomula; M, adult males.

Briefly, miRNA profiles at several stages of *S. japonicum* development have been characterized; miRNAs present in lung-stage schistosomula, hepatic-stage schistosomula, adult males, adult females, and eggs of this species were documented, and 199 novel miRNAs with stage specificities were found (Cai et al., 2011). Additional research evaluating miRNA profiles in *S. japonicum* eggs have indicated that sja-miR-71b-5p, sja-miR-71, sja-miR-1, sja-miR-36-3p, and sja-miR-124-3p were the most abundant miRNAs at this developmental stage (Cai et al., 2013a). Concurrent with research in *S. japonicum*, 112 miRNAs (including 84 novel miRNA families) were reported in adult worms of *S. mansoni* (Marco et al., 2013). Among them, 26 miRNAs were shown to be conserved in other flatworms such as *Schmidtea mediterranea* and *Dugesia japonica* (Marco et al., 2013). In addition, a more recent study reported a total of 2258 miRNAs identified in *S. japonicum* collected from single- and double-sex infected mice (Sun et al., 2014); however, the authors did not provide information about the number of evolutionary conserved miRNAs. Moreover, in our recent work, five pathogen-specific miRNAs were identified in the plasma of rabbits infected with *S. japonicum*, including four known miRNAs (Bantam, miR-3479, miR-10, and miR-3096), and one novel miRNA (sja-miR-8185) (Cheng et al., 2013).

On comparing these known miRNAs across the Platyhelminthes, most were found to be conserved (Jin et al., 2013). In addition, some *S. japonicum* miRNAs (e.g., miR-1b, miR-124, miR-190, et al.) also displayed evolutionary conservation among *Homo sapiens, Mus musculus, Caenorhabditis elegans*, and *Drosophila melanogaster* (Cheng and Jin, 2012).

## Potential role of *Schistosoma* miRNAs in worm development and the pathogenesis of schistosomiasis

Different stages of schistosome development are associated with different categories of miRNAs, suggesting that miRNAs may be involved in the regulation of schistosome development (Krautz-Peterson and Skelly, 2008; Xue et al., 2008; Chen et al., 2010; Hao et al., 2010; Luo et al., 2010; Cai et al., 2011; Simoes et al., 2011). Several lines of evidence also indirectly imply that miRNAs may regulate the pathogenesis of schistosomiasis (Cheng et al., 2013; Hoy et al., 2014).

First, the key components of miRNA biogenesis, including the Ago proteins (Chen et al., 2010) and Dicer (Krautz-Peterson and Skelly, 2008; Luo et al., 2010), have been shown to be differentially expressed during different stages of schistosome development. Second, some of miRNAs such as miR-36, miR-71, bantam, miR-7, and others, have been shown to specifically express in a specific stage of *S. japonicum* (Xue et al., 2008; Hao et al., 2010; Cai et al., 2011). In addition, the similar phenomenon was also observed in *S. mansoni* (miR-4, miR-6, miR-9, miR-32, miR-125, miR-3, and miR-5 were expressed in adult worms only, and miR-20, miR-18, miR-22, miR-26, and bantam were expressed in schistosomula only) (Simoes et al., 2011). Third, bioinformatic analyses have indicated that several evolutionarily conserved miRNAs (miR-8, miR-1, miR-124, miR-71, and miR-195) in schistosomes may regulate phylogenetically conserved mRNA targets (Cheng and Jin, 2012); however, experimental confirmation of these results is needed.

**Figure 1:**
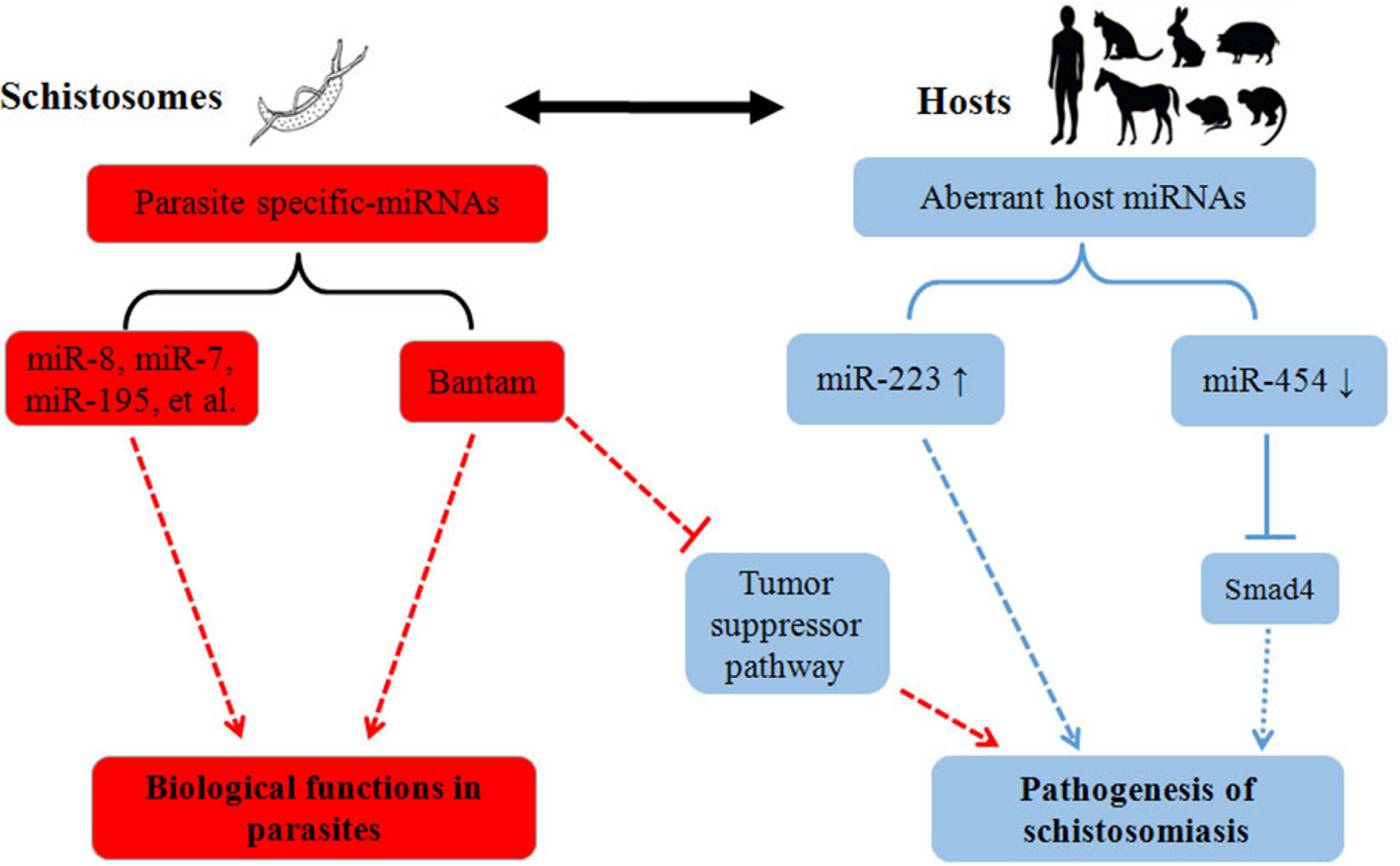
**Summary of the roles of miRNAs in the pathogenesis of schistosomiasis**. Parasite activities are depicted in red, and host activities are depicted in blue. The solid lines indicate the proven biological effects of miRNAs, and the dotted lines indicate their putative effects.

Besides role of miRNAs in schistosome development, recent work within our laboratory implies that schistosome-derived miRNAs may regulate the pathogenesis of schistosomiasis within a final host (Cheng et al., 2013). In this study, we identified *Schistosoma*-specific Bantam miRNA within plasma derived from a host infected with *S. japonicum*. In *Drosophila*, it has been shown that Bantam can target a tumor-suppressor pathway, and that overexpression of Bantam led to cellular growth and the suppression of cellular death (Nolo et al., 2006). In addition, it was reported that human schistosomiasis is associated with increased risks for bladder (*S. haematobium*) and liver (*S. mansoni, S. japonicum*) cancers (Takemura et al., 1998; Gryseels et al., 2006). Given this research, we hypothesized that schistosome-specific miRNAs, such as Bantam, may be involved in the etiology of cancers in a host infected with schistosomes (Figure 1); however, further studies will need to be carried out to test this hypothesis.

## Role of host miRNAs in the pathogenesis of schistosomiasis

In addition to schistosome-derived miRNAs, recent studies also suggest that host miRNAs may be involved in regulating the pathogenesis of schistosomiasis (Figure 1). Integrative analysis of miRNA and mRNA profiles in murine liver suggested that miRNAs, and their corresponding mRNA targets, may function coordinately to regulate the hepatic pathology of a host during schistosome infection (Cai et al., 2013b). Furthermore, miRNA profiles in different tissues (liver, spleen, and lung) of *S. japonicum*-infected mice implied that miRNAs may be involved in the regulation of several signaling pathways—such as the MAPK, insulin, Toll-like receptor, and TGF-β pathways—during schistosome infection (Han et al., 2013). In a recent study, miR-223 was primarily found in Kupffer cells in the liver, and the expression of this miRNA was dramatically elevated in liver cells of schistosome-infected mice (He et al., 2013); it is possible that the expression levels of miR-223 could reflect the extent of pathological changes in the liver of an infected host. Moreover, miR-454, a target miRNA of *Smad4*, was found to be down-regulated in *S. japonicum*-induced murine liver fibrosis models, while α-SMA and Smad4 were up-regulated (Zhu et al., 2014); these results suggested that the down-regulation of miR-454 may be involved in the pathogenesis of liver fibrosis in *S. japonicum*-infected mice. Despite these findings, the functions of miR-223 and miR-454 need to be further investigated at different biological levels, in order to fully understand the roles that these miRNAs play in the pathogenesis of schistosomiasis.

Since certain miRNAs have been shown to potentially play an important role in the pathogenesis of schistosomiasis, the usage of the antisense technology blocking specific miRNAs and/or an augment method for improving the expressions of some miRNAs may have a potential to provide alternative strategies for schistosomiasis control as optimism have been demonstrated from miRNA-based anti-tumor therapy (Krutzfeldt et al., 2005; Ma et al., 2010; Wang et al., 2012a).

## Role of miRNAs in schistosomiasis diagnosis

Early diagnosis of schistosomiasis is critical to control this disease. However, no reliable biomarker exists for early diagnosis of schistosomiasis. Circulating miRNAs, which are present in a stable form in the plasma or serum of an infected host, have been considered ideal biomarkers for the diagnosis of some cancers. It is possible that such circulating miRNAs could also serve as biomarkers for schistosomiasis diagnosis.

As mentioned above, five pathogen-specific miRNAs in the circulation of a *S. japonicum*-infected host were identified in our study and the subsequent validation study indicated that there is a correlation between the abundance of schistosome-specific miRNAs in the plasma of schistosome-infected mice and the number of *S. japonicum* cercariae in the inoculum (Cheng et al., 2013). More recently, eleven schistosome-specific miRNAs were identified in mice infected with *S. mansoni* (Hoy et al., 2014); three of these parasite-derived miRNAs (Bantam, miR-3479-3p, and miR-277) may be potential biomarkers for schistosomiasis diagnosis. Similarly, we detected three miRNAs (Bantam, miR-3479, and miR-10) in the plasma of mice infected with *S. japonicum* (Cheng et al., 2013). Collectively, these results suggest that parasite-specific miRNAs in the circulation of a host may be potential biomarkers for schistosomiasis diagnosis. However, it remains to be determined whether this kind of pathogen specific circulating miRNAs is associated with specific species in schistosomes.

Apart from *Schistosoma*-specific miRNAs, aberrant host miRNAs associated with schistosome infection may also be potential indicators of schistosomiasis. A recent study indicated that miR-223 was significantly up-regulated in the serum of mice infected with *S. japonicum* and returned to near normal levels on praziquantel treatment (He et al., 2013), implying that up-regulated murine miR-223 may be a biomarker for *Schistosoma* infection. However, the possibility that altered serum levels of miR-223 are caused by other diseases or the pathogenesis of other organs needs to be assessed.

Currently, circulating miRNA detection mostly relies on qRT-PCR (Cheng, 2014). Additionally, several recent studies also documented that the sensitivity of miRNA detection could be significantly improved by using novel target miRNA amplification methods such as RAKE assay (Nelson et al., 2004), rolling-circle amplification (Cheng et al., 2009), and DNA concatamers-based amplification (Hong et al., 2013) and also the combination of new signal detection techniques including a total internal reflection fluorescence microscope (Ho et al., 2014) or an electrochemical device (Hong et al., 2013; Ren et al., 2013). In future, it may be necessary to evaluate these methods for schistosomiasis diagnosis by detecting circulating miRNAs.

## Summary

Recent studies have indicated that miRNAs play important roles in schistosome development and their parasitism. Although analyses of miRNA profiles have provided valuable information regarding the mechanisms underlying the pathogenesis of schistosomiasis, and suggested potential novel applications in diagnostics, our current understanding of these molecular mechanisms remains limited. Consequently, it is necessary to characterize the functions of schistosome-specific miRNAs and clarify their potential as diagnostic markers for schistosomiasis. Since miRNAs exert their effect by targeting protein-encoding mRNAs, verifying miRNA targets in both hosts and parasites is essential for understanding the mechanisms of host-parasite interactions, and developing novel therapeutic agents for schistosomiasis.

### Conflict of interest statement

The authors declare that the research was conducted in the absence of any commercial or financial relationships that could be construed as a potential conflict of interest.
